# Commentary: Interprofessional Collaboration and Involvement of Parents in the Management of Painful Procedures in Newborns

**DOI:** 10.3389/fped.2020.599759

**Published:** 2020-11-05

**Authors:** Mio Ozawa

**Affiliations:** Division of Nursing Sciences, Graduate School of Biomedical & Health Sciences, University of Hiroshima, Hiroshima, Japan

**Keywords:** parent, readiness, pain, parental involvement, neonatal intensive care unit, pain management

## Introduction

The article by Balice-Bourgouis et al. entitled “Interprofessional Collaboration and Involvement of Parents in the Management of Painful Procedures in Newborns” (1) showed feasibility and acceptability of the NEODOL (NEOnato DOLore) intervention (2) to improve interprofessional collaboration, including parents as active care providers in neonatal pain management. Parental involvement in neonatal pain management, including both parental perception of their infant's pain and their satisfaction with pain practices in NICUs, is a relatively new area of research (3). In the early days of neonatal intensive care, the focus was on hygiene and infection control. Families were seen as dangerous potential sources of contamination, and newborns were considered to be neither in need of human relationships nor able suffer long-term consequences from early life experiences. To date, researchers have begun evaluating the impact of parental involvement in their infant's non-pharmacological or pharmacological pain management for procedural or postoperative pain using randomized controlled trials (4–7). The latest systematic review of acute procedural pain management guidelines for neonates has suggested that recommendations need to involve not only pharmacological and non-pharmacological pain treatment but also parental and interprofessional collaboration (8). In addition, this article (1) and other previous (9, 10) studies demonstrated parents' desire for knowledge about infant pain. When parents are educated either verbally or with demonstrations about specific interventions, they have shown they will effectively employ the pain management or reduction interventions during subsequent painful procedures for their infants (1, 9, 10). Therefore, there is a possibility that parental presence and provision of pain care, or working with health-care providers to advocate for pain management in their infants, will become a mainstream practice in NICUs. From such a background, the NEODOL is advanced and good initiative.

However, parents' participation in infant pain management was not popular with health-care professionals in NICU. Greisen et al. (11) investigated policies regarding families visiting NICUs in eight European countries and suggested that unrestricted parental presence is not yet a uniformly accepted standard among European NICUs, and that parental presence is often restricted during medical rounds and procedures. Regarding pain management, a national survey showed that only 11% of Japanese NICUs involved parents in pain management (12), and French NICU mothers reported that they received some information on infants' pain, but only 23% perceived the information they received as sufficient (9).

## Negative Parental Views About Parental Participation in Pain Management

On the other hand, negative parental views about parental presence and parental provision of pain care for their infants has also been established in this article (1). Frank et al. (3) showed the roles parents wanted to play in infant pain management ranged from none (not presence or viewed infant comfort as the role of only the nurse or doctor) to advocating for the infant (believing this was their primary responsibility, in partnership with clinical care team). Other previous study suggested that mother's memories of their infant's pain may be associated with later post-traumatic stress symptoms (13); further research is needed to investigate parents' experience of their infant's pain, including negative and positive impact. In addition, the previous study clearly demonstrated that the cultural, ethical, and legal values of the country or region where the study took place was the strongest descriptive variable predicting the parental visits in the NICUs (9, 10, 12). The latest meta-synthesis of qualitative studies also suggested that parental stress and anxiety regarding infant's pain was one of the factors that affected parental participation in pain management in NICU; parents were both concerned that their infants were in pain and were frightened of watching painful procedures (14). Therefore, health-care professionals must consider parental stress and anxiety when working with parents to educate them regarding pain management for infants.

## Parental Readiness in Neonatal Intensive Care Unit

According to the Oxford dictionary, readiness is defined as the state of being ready or prepared. To date, while there are some studies about NICU parental readiness for their children's discharge from the hospital (15–17), the previous studies have clearly described the physical and psychological state of the parent, the state of the child, knowledge, confident, a technique, and the family budget for the element of parents' readiness for the child's discharge (15–17). However, there is limited evidence regarding parental readiness for participating in the pain management of their infant in the NICU. The latest meta-synthesis focused on factors that influence parental participation in pain management in NICU suggested parental readiness to participate was not clear (14). Nurses must be teachers and coaches based on patient readiness. Therefore, health-care professionals and researchers need to explore the parental readiness for participating in their infant's pain care in NICUs to improve patient- and family-centered neonatal pain care. Deep understanding of the parental readiness seeks to lay the ground work for future studies regarding the role of parents in interventions for their infants in the NICU.

## Discussion

In short, when the authors explore the NEODOL intervention following the next phase (three) on the revised the Medical Research Council (MRC) guidelines (18), which is a framework to help researchers and research funders to recognize and adopt appropriate method in developing and evaluating complex intervention, they should consider the parental readiness for being involved in their infants' pain care. That may further improve the NEODOL intervention.

## Author Contributions

The author confirms being the sole contributor of this work and approved it for publication.

## Conflict of Interest

The author declares that the research was conducted in the absence of any commercial or financial relationships that could be construed as a potential conflict of interest.
